# Changing trends in lymphoid neoplasm distribution in South Korea: analysis of 8615 cases from a single institute, 1997–2016: An observational study

**DOI:** 10.1097/MD.0000000000017641

**Published:** 2019-11-11

**Authors:** Jongmin Sim, Takuya Takayama, Junhun Cho, Seok Jin Kim, Won Seog Kim, Howe J. Ree, Young Hyeh Ko

**Affiliations:** a Department of Pathology and Translational Genomics; b Division of Hematology-Oncology, Department of Internal Medicine, Samsung Medical Center, Sungkyunkwan University School of Medicine, Seoul, Korea; c University of the Ryukyus School of Medicine, Okinawa, Japan.

**Keywords:** epidemiology, hematologic neoplasms, incidence, Korea

## Abstract

The purpose of this study was to evaluate changes in the proportion of lymphoid neoplasm subtypes in South Korea. A total of 8615 cases of lymphoid neoplasms diagnosed in 1997–2016 at Samsung Medical Center in South Korea were classified according to the 2008 World Health Organization system. The total number and proportion of lymphoid neoplasms were compared between these two decades, with data from nationwide studies, and with other countries. To evaluate changes in the proportion of subtypes, crude rate of each subtype per 100 lymphoma patients during each decade and age adjusted rate were calculated. There were 3024 patients with lymphoid neoplasm in 1997–2006, and 5591 in 2007–2016, which represents an average increase of 1.85 times over the 20-year study period. Crude rate and age adjusted rate were increased in Hodgkin's lymphoma and mature B cell lymphoma while precursor lymphoid neoplasms and mature T cell lymphoma were decreased. Among B cell neoplasms, age adjusted rate of plasma cell neoplasm, follicular lymphoma, mantle cell lymphoma increased while there was no significant change in extranodal marginal zone lymphoma and Burkitt lymphoma. The increase in follicular lymphoma was due to the increases in nodal follicular lymphoma of low grade and duodenal-type follicular lymphoma. These results are consistent with the dynamics of causative factors, including socioeconomic factors, in Korea.

## Abstract 2

Supplemental Digital Content is available in the text

## 1 Introduction

Lymphoid malignancy is a heterogeneous cancer group consisting of neoplastic lymphoid cells showing various morphologic and immunophenotypic findings corresponding to lymphocytes at various stages of lymphocyte differentiation. The origin, etiology, clinical features, and response to therapy of lymphoid neoplasms are highly diverse. The World Health Organization (WHO) classification recognizes more than 50 lymphoid neoplasm subtypes based on their unique clinical, histologic, immunophenotypic, and genetic findings.^[1,2]^

Lymphoid malignancy represents 8.3% of all cancers, according to 2018 SEER data.^[3]^ In Korea, lymphoid malignancies account for 65%∼70% of all blood cancers,^[4,5]^ with approximately 8900 new patients and 4100 deaths annually.^[5,6]^ Currently, the incidence of lymphoid tumors is gradually increasing in both Western and Asian countries, possibly attributable to socioeconomic and environmental factors that can modulate immune function and increase exposure to carcinogens.^[7–9]^ Korea has been through industrialization from the 1960s to the 1980s and achieved a significant degree of socioeconomic improvement similar to that of other developed countries, resulted in considerable changes in quality of life, eating habits, and population distribution.^[10]^ Such social and environmental changes may also lead to changes in the incidence of lymphoid neoplasms and their subtypes. It is well known that subtype incidences of malignant lymphoma differ according to geographic region, as well as among countries in the same geographic region.^[11–15]^ Compared with Western countries, Asian countries have been reported to have higher rates of T/NK-cell neoplasms and lower incidences of follicular lymphoma and Hodgkin lymphoma. Epstein–Barr virus (EBV)-associated extranodal NK/T cell lymphoma, nasal type, is more frequent in Korea and China compared with Japan, where Human T-cell leukemia virus type 1 (HTLV-1)-associated adult T cell leukemia is prevalent.^[16–19]^ In Korea, Helicobacter-associated extranodal marginal zone lymphoma of mucosa-associated lymphoid tissue (ENMZL) is a common lymphoid neoplasm subtype.^[17,18]^

Regarding the incidence of lymphoid neoplasm, some Korean studies using multicenter data have reported the incidence of lymphoid neoplasm of Korea, although these data have limitations, including the omission of liquid-phase lymphoid neoplasm.^[8,12,18]^ The most recent data are based on a nationwide hospital-based cancer registry that covers all lymphoid malignancies, both liquid and solid phase, for 1999–2012. These data showed continuously increasing incidence of all lymphoid malignancies.^[8]^ Two studies reported that follicular lymphoma is decreasing,^[17,18]^ while another showed the opposite.^[8]^ However, the frequency of extranodal NK/T-cell lymphoma was also reported in these two studies to be decreasing.^[17,18]^

To clarify the changing trends in the frequency of lymphoid neoplasm subtypes in Korea, we analyzed 8615 cases of lymphoid neoplasms diagnosed over 20 years at a single hospital. While our data are limited by having come from a single institution, relative to other studies, they also have the advantage that every case was reviewed by the authors (J Sim, YH Ko, HJ Ree) and included a large number of cases, representing 10% of the total lymphoma cases in the nation since 2012.

## 2 Materials and methods

### 2.1 Patients and tissue samples

All lymphoid neoplasms diagnosed in the solid organ at the Samsung Medical Center in 1997–2016 were retrieved from surgical pathology medical records in the department of pathology database using search terms “lymphoma,” “lymphoproliferative disease,” “lymphoproliferative disorders,” “plasmacytoma,” and “myeloma.” All cases of lymphoid leukemia, lymphoma, and myeloma diagnosed in bone marrow and blood samples were also retrieved from the hospital medical records using ICD-O codes. All cases were diagnosed according to the 2008 WHO classification system, based on morphological, immunophenotypical, and clinical features. Cases diagnosed prior to the 2008 WHO classification system were reclassified according to the 2008 classification by the authors (J Sim, YH Ko, HJ Ree). Posttransplantation lymphoproliferative disorders and cases with insufficient immunohistochemical evidence to allow 2008 WHO classification were excluded. Only the initial diagnosis for each patient was included. A total of 8615 patients diagnosed with lymphoid neoplasms in 1997–2016 were enrolled. Age, gender, pathological diagnoses, and source of specimen were also obtained from the medical records. The study was approved by the Institutional Review Board of Samsung Medical Center (IRB File No. 2017-08-148-001).

### 2.2 Ancillary studies for diagnosis

For 2008 WHO classification, immunohistochemical staining was performed at diagnosis in all cases of lymphoma/plasma cell neoplasms diagnosed in solid organ and body fluid. Basically, immunostaining for B- and T-cell markers (CD3 [Dako, Santa Clara, CA, USA] and CD20 [Leica, Wetzlar, Germany]) were performed in all cases. In cases requiring further immunophenotyping, additional markers such as CD1a, CD2, CD4 (Leica), CD5, CD7, CD8 (Leica), CD10 (Leica), CD15, CD21, CD23, CD30, CD34, CD56, CD57, CD68, CD79a, CD99, CD123, CD138, epithelial membrane antigen, cyclin D1, sox11, bcl2, bcl6, multiple myeloma oncogene 1 (MUM-1), PAX-5, TCR-βF1, TCR-cγM1, T-cell restricted intracellular antigen-1 (TIA-1) (Immunotech, Marseille, France), granzyme B, ALK-1, myeloperoxidase, IgM, IgG, IgA, IgD (Thermo scientific, Waltham, MA, USA), kappa light chain, lambda light chain, (Dako), HHV-8, and terminal deoxyribonucleotidyl transferase (Dako) were used. To diagnose myeloma and leukemia in bone marrow, flow cytometric immunophenotyping was performed. Acute leukemia panel included cCD3, CD10, CD11c, CD13, CD14, CD19, CD20, cCD22, CD33, CD34, CD38, CD64, CD66c, cCD79a, CD117, cMPO, nTdT, and CD45. Multiple myeloma panel included CD19, CD38, CD138, CD45, CD56, CD117, CD28, kappa light chain, and lambda light chain.

EBV was detected by in situ hybridization using EBER (EBV-encoded small nuclear small RNA) and quantitative EBV DNA analysis from blood samples. Clonality analysis was carried out using conventional PCR or BIOMED-2 Multiplex PCR for IgH gene, TCR-γ, and β gene.^[20]^ When necessary, C-MYC and Bcl-2 translocation were detected by fluorescence in situ hybridization (FISH).

### 2.3 Statistical analyses

Crude rate for proportion of each subtype was determined as a frequency rate per 100 lymphoma patients during 1997–2006 and 2007–2016. Calculation of age adjusted rate used Korea's standard population in the year of 2000 (Statistics Korea, http://kostat.go.kr/portal/korea/index.action).

## 3 Results

### 3.1 Demographics

The cases were 3740 females and 4875 male patients (M:F ratio 1.3:1) with a median age of 54 years (range: 0–96). Biopsy sites were lymph node (1774 cases, 20.6%), bone marrow (2233 cases, 25.9%), and extranodal sites (4608 cases, 53.5%), including (in descending frequency) stomach (1229 cases, 14.4%), upper aerodigestive tract (480 cases, 5.6%), intestine (478 cases, 5.5%), brain (308 cases, 3.6%), skin (256 cases, 3%), Waldeyer's ring (239 cases, 2.8%), and bone (208 cases, 2.4%) (Table 1; Supplementary Table 1).

**Table 1:**
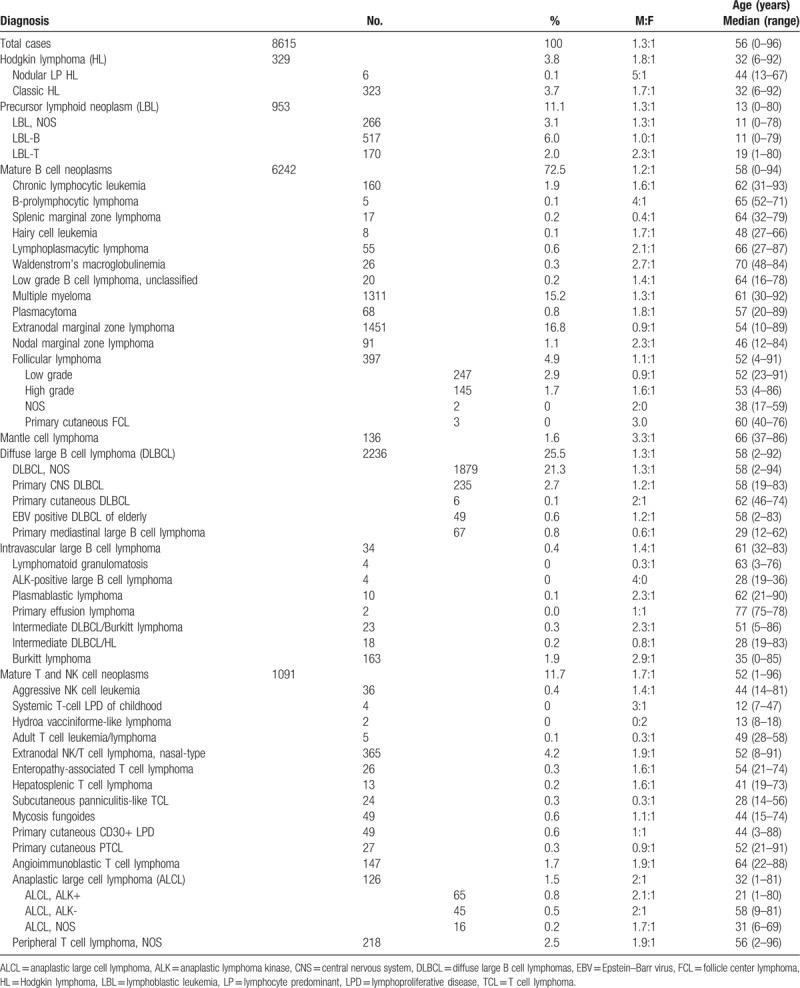
Distribution of histologic subtypes, age, and sex of 8615 patients with lymphoid neoplasm diagnosed between 1997 and 2016.

### 3.2 Subtype distribution of all lymphoid neoplasms

**Figure 1:**
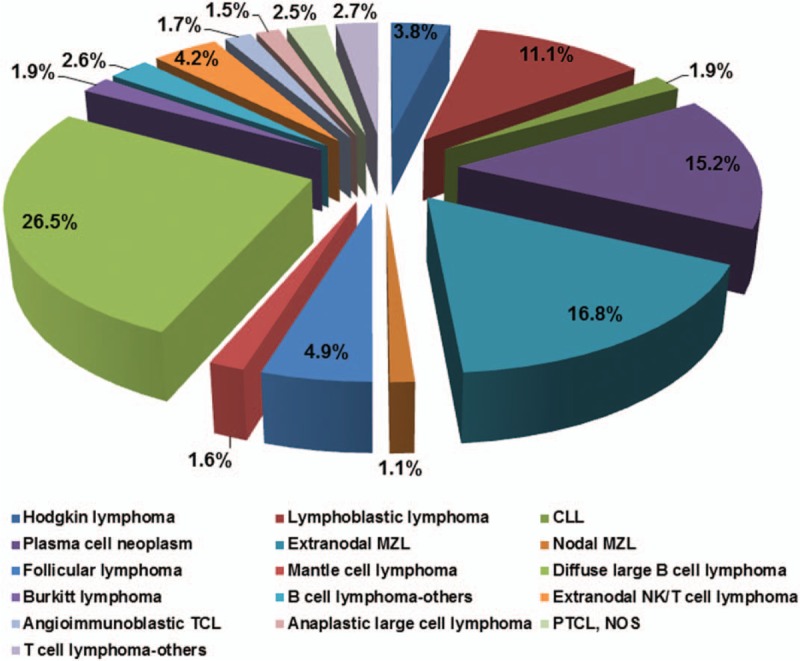
Distribution of histologic subtypes of 8615 patients with lymphoid neoplasm diagnosed between 1997 and 2016. CLL = chronic lymphocytic leukemia/small lymphocytic lymphoma, extranodal MZL = extranodal marginal zone lymphoma of mucosal-associated lymphoid tissue, MZL = marginal zone lymphoma, PTCL, NOS = peripheral T cell lymphoma, not otherwise specified, TCL = T cell lymphoma.

The 8615 cases were classified as Hodgkin lymphoma (329 cases, 3.8%), precursor lymphoid neoplasms (953 cases, 11.1%), mature B cell neoplasms (6242 cases, 72.5%), and mature T and NK cell neoplasms (1091 cases, 11.7%). All but six of the 329 Hodgkin lymphomas were classic type. Among the mature B cell neoplasms, frequent subtypes included (in descending frequency) diffuse large B cell lymphoma (2236 cases, 26.5%), ENMZL (1451 cases, 16.8%), plasma cell neoplasms (1379 cases, 15.2%), follicular lymphoma (397 cases, 4.9%), chronic lymphocytic leukemia/small lymphocytic lymphoma (CLL/SLL) (160 cases, 1.9%), Burkitt lymphoma (163 cases, 1.9%), and mantle cell lymphoma (136, 1.6%). Among the mature T and NK cell neoplasms, extranodal NK/T cell lymphoma, nasal-type, was the most common subtype, accounting for 4.2% (365 cases) of all lymphoid neoplasms, followed by peripheral T cell lymphoma, not otherwise specified (PTCL, NOS) (218 cases, 2.5%), angioimmunoblastic T cell lymphoma (147 cases, 1.7%), and anaplastic large cell lymphoma (126 cases, 1.5%) (Table 1; Fig. 1).

### 3.3 Subtype distribution according to age and gender

Most lymphoid neoplasm subtypes were male predominant, except for splenic marginal zone lymphoma (MZL) (M:F = 0.4:1), ENMZL (M:F = 0.9:1), primary mediastinal large B cell lymphoma (M:F = 0.6:1), subcutaneous panniculitis-like T cell lymphoma (M:F = 0.3:1), and primary cutaneous T cell lymphoma, NOS (M:F = 0.9:1). Median patient age was 32 years (range: 6–92) in Hodgkin lymphoma, 15 years (range: 0–80) in lymphoblastic neoplasms, 58 years (range: 0–94) in mature B cell neoplasms, and 52 years (range: 1–96) in mature T cell neoplasms (Table 1).

According to age grouping, the most frequent subtype was lymphoblastic neoplasms in the first and second decades and diffuse large B cell lymphoma and Hodgkin lymphoma in the third decade. Subsequently, diffuse large B cell lymphoma was the most frequent subtype among adults and older adults. Extranodal marginal zone lymphoma was the second most frequent subtype among those in their 40s, 50s, and 60s, while among those in their 70s to 90s, plasma cell neoplasms were the second most common subtype (Supplementary Table 2). Distribution of predominant subtype differed based on gender. Burkitt lymphoma is more prevalent among boys, so in the first decade, male Burkitt lymphoma was the second most common lymphoma, followed by lymphoblastic leukemia/lymphoma (but not in girls). Likewise, ENMZL showed a slight female predominance; thus, in their 40s and 50s, ENMZL was the most frequent subtype among female patients (but not males) (Supplementary Tables 3 and 4).

### 3.4 Subtype distribution according to biopsy site

Subtype distribution differed according to site. In lymph nodes, the most frequent subtypes (in descending frequency) were diffuse large B cell lymphoma (n = 588, 33.1%), follicular lymphoma (n = 268, 15.1%), and Hodgkin lymphoma (n = 243, 13.7%). In bone marrow, plasma cell myeloma was the most common subtype, accounting for 48.2% of 2233 cases, followed by lymphoblastic neoplasms, accounting for 34.9%. In the extranodal sites, diffuse large B cell lymphoma (n = 1586, 35.8%) and ENMZL (n = 1437, 32.5%) were the predominant subtypes, followed by extranodal NK/T cell lymphoma (n = 350, 7.9%), plasma cell neoplasm (n = 145, 3.3%), follicular lymphoma (n = 126, 2.8%), and PTCL, NOS (n = 102, 2.3%). In the stomach, ENMZL (n = 891, 72.5%) and diffuse large B cell lymphoma (n = 269, 21.9%) were the major subtypes. Diffuse large B cell lymphoma (n = 50, 31.6%), lymphoblastic lymphoma (n = 48, 30.4%), and Hodgkin lymphoma (n = 39, 24.7%) were prominent subtypes in the mediastinum. In the eye, ENMZL accounted for 89.6% of 280 cases. In the nasal cavity, extranodal NK/T cell lymphoma (n = 221, 75.7%), diffuse large B cell lymphoma (n = 36, 12.3%), and plasma cell neoplasm (n = 17, 5.8%) were the most common subtypes (Supplementary Table 1).

### 3.5 Changing trends in the relative frequency of subtypes among lymphoid neoplasms

#### 3.5.1 Change in the number of patients with lymphoid neoplasms

We evaluated changes in the number of patients and relative frequency of lymphoma subtypes over two decades (Table 2). The number of patients who have visited the Samsung Medical Center since 1997 has continued to increase. In 1997–2006, the number of patients with lymphoid neoplasm was 3024, and 5591 in 2007–2016, that is, an average increase of 1.85 times over the 20-year study period. Increased rates were higher in Hodgkin lymphoma (2.16 times) and mature B cell lymphoma (2.03 times), but changes in rates of precursor lymphoid neoplasms and mature T cell lymphoma did not reach the average increase (1.41 and 1.36 times, respectively). Among B cell lymphomas, remarkably, the increase in CLL/SLL, plasma cell neoplasms, follicular lymphoma, and mantle cell lymphoma, especially follicular lymphoma more than doubled, exceeding the average rate of increase, while increase in Burkitt lymphoma was minimal (1.2 times). ENMZL showed the average rate of increase. Among mature T cell lymphomas, the number of patients with angioimmunoblastic T cell lymphoma increased 2.27 times; however, among those with other types of T cell lymphomas, the rate of increase was lower than average.

**Table 2:**
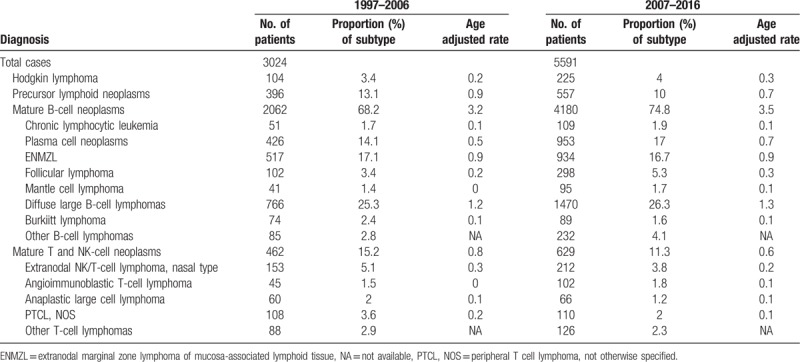
Changes of major subtype distribution of lymphoid neoplasms over past 20 years.

#### 3.5.2 Change in the proportion of subtypes among all lymphoid neoplasms

To evaluate changes in the proportion of subtypes, crude rate of each subtype per 100 lymphoma patients during each decade and age adjusted rate were calculated. Crude rate and age adjusted rate were increased in Hodgkin's lymphoma and mature B cell lymphoma while precursor lymphoid neoplasms and mature T cell lymphoma were decreased (Table 2).

Among B cell neoplasms, age adjusted rate of plasma cell neoplasm, follicular lymphoma, mantle cell lymphoma increased while there was no significant change in extranodal marginal zone lymphoma and Burkitt lymphoma. The increase in low-grade follicular lymphoma was ascribed to an increase in both nodal follicular lymphoma of low grade and to duodenal-type follicular lymphoma (data not shown). Among mature T and NK cell lymphomas, the proportions of subtypes decreased, except for angioimmunoblastic T cell lymphoma.

#### 3.5.3 Comparisons with national data

Total numbers and proportions of major lymphoid neoplasm subtypes in our hospital were compared with data from previously published nationwide studies, initiated by the Hematolymphoid Study Group of the Korean Society of Pathologists (KSP) and the Korean National Cancer Incidence Database (KNCIDB) based on the national population-based cancer-registry program initiated by the Korean Ministry of Health and Welfare. As shown in Table 3, each dataset has confounds. KSP data include very low number of plasma cell neoplasms and precursor cell neoplasms, which are mainly diagnosed in bone marrow. KNCIDB data contain a high number of undetermined type of lymphoid neoplasms in which immunophenotype information is insufficient for diagnosis according to the WHO classification. Compared with the KNCIDB data, rates from our hospital are similar for precursor cell neoplasms, plasma cell neoplasms, and mature T/NK cell neoplasms, while rates of ENMZL and follicular lymphoma are higher. Compared with KSP data, the proportion of T/NK cell neoplasm at our hospital was significantly lower.

**Table 3:**
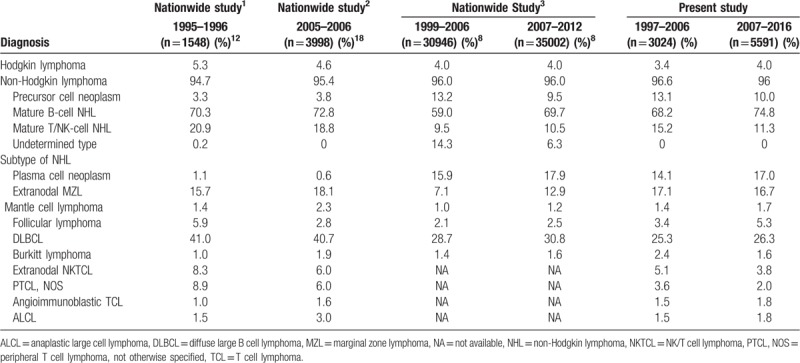
Comparison with Nationwide studies in Korea performed previously.

#### 3.5.4 Comparison with data from other countries

Comparing our data for 2007–2016 with those from Japan and China for 2001–2006 and 2004–2008, respectively, our cohort showed a markedly lower proportion of T/NK cell neoplasm (11.3% vs 25.5% and 26.4%, respectively). The frequency of extranodal NK/T cell lymphoma was highest in China (11%) followed by our institution (3.8%) and Japan (1.6%). When comparing our data for 2007–2016 with that from the USA in 2016, our cohort showed a similar frequency of Hodgkin lymphoma (4.0% vs 6.2%), diffuse large B cell lymphoma (26.3% vs 20.2%), and plasma cell neoplasms (17% vs 19%), but markedly lower proportions of CLL/SLL (1.9% vs 15.3%) and follicular lymphoma (5.3% vs 10.2%) and higher proportions of T/NK cell lymphoma (11.3% vs 6.1%) and ENMZL (16.7% vs 3.2%) (Table 4).

**Table 4:**
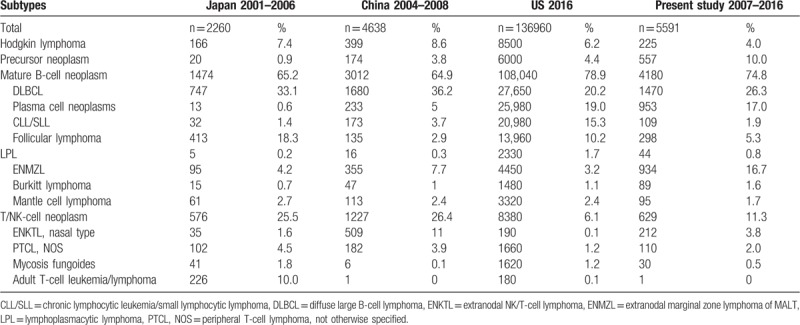
Comparison of subtype frequency with the data from other countries.

## 4 Discussion

The etiology of malignant lymphoma is multifactorial, including genetic factors, infectious agents, autoimmune diseases, and socioeconomic factors. The International Lymphoma Epidemiology Consortium (InterLymph) project identified numerous environmental, lifestyle, medical, and genetic risk factors by examining pooled studies of epidemiological surveys and single nucleotide polymorphisms.^[21]^ Family history of hematologic malignancy, autoimmune diseases, atopic conditions, lifestyle factors (e.g., smoking, alcohol, anthropometric measures, use of hair dye), and sun exposure are associated with non-Hodgkin's lymphoma risk.^[22–24]^ These studies also revealed etiologic commonality and heterogeneity among non-Hodgkin's lymphoma risk subtypes. Family history of hematologic malignancy, autoimmune diseases, atopic conditions, and alcohol consumption are associated with risk or prevention across several subtypes.^[21]^ Eczema, T cell-activating autoimmune diseases (e.g., celiac), and cigarette smoking were more strongly associated with risk of PTCL and/or mycosis fungoides/Sezary syndrome, whereas hepatitis V virus infection, blood transfusion, and B cell-activating autoimmune disease were more strongly associated with B cell lymphomas.^[25]^

According to nationwide studies, malignant lymphoma in Korea is characterized by a lower frequency of Hodgkin lymphoma, follicular lymphoma, and CLL/SLL compared with Western populations.^[12,18]^ Previous study from United States analyzing incidence rate differences of malignant lymphoma by birthplace and acculturation demonstrated that the incidence rates were significantly lower in foreign-born Asian than US-born Asian patients for certain lymphoma subtypes, specifically CLL/SLL, follicular lymphoma, and nodular sclerosis Hodgkin lymphoma. This data strongly suggests a role of environmental factors that influence the risk of FL and CLL/SLL,^[26]^ while risk variants identified by genome-wide association study does not exclude a role for genetic susceptibility to follicular lymphoma and CLL/SLL.^[27,28]^ Infectious agents are important factors in the characterization of lymphomas in Koreans, particularly the higher incidence of EBV-positive extranodal NK/T cell lymphoma might be associated with primary EBV infection at early childhood and higher prevalence of EBV infection. In one study reported in 1994, 90% of children aged 7–9, and 100% of children aged 10–15 years had EBV antibodies.^[29]^ High frequency of gastric MZL appears to ascribe to a high prevalence of *Helicobacter pylori* infection among Koreans.^[30,31]^

As domestic socioeconomic and health care conditions have improved markedly over the last four decades in Korea, there have been changes in the epidemiology of infectious diseases, the population structure by age, and lifestyles. Due to significantly increased life expectancy, the elderly population has also increased dramatically, while the birth rate has been steadily declining. Obesity is increasing every year, and this has become a very important public health issue.^[32]^ The seroprevalence of EBV infection in young adults and adolescents was 100% in 1994, but decreased to 87.2% in 2007.^[33]^ Likewise, the seroprevalence of H. pylori in healthy adults has gradually decreased, from 66.9% in 1998 to 54.4% in 2011.^[34]^ Although well-planned epidemiologic evaluation correlating lymphoma subtypes and etiologic factors has not been carried out in Korea, we can expect that such health and socioeconomic changes would result in changes in the subtypes of lymphoid neoplasms.

The occurrence of cancer is increasing because of population growth and aging, as well as increasing prevalence rates of established risk factors such as smoking, overweight, physical inactivity, and changing reproductive patterns associated with urbanization and economic development.^[35]^ In the USA, lymphoma incidence rates increased steadily during the 1970s and 1980s, leveled off in the 1990s, and have declined slightly (0.3% per year) since 2001 in females and since 2004 in males.^[36,37]^ In Korea, patients with malignant lymphoid neoplasm are continuously increasing. According to KNCIDB data, registered patients with lymphoid neoplasm (in 2002–2012) increased 1.84 times (3606 in 2002 and 6638 in 2012). The overall age-standardized incidence rates of all lymphoid malignancies increased from 6.9 to 9.9, with an annual percentage change of 3.2% between 1999 and 2012.^[8]^ Accordingly, lymphoma patients in our cohort increased 1.85 times during the 20-year study period. The most notable change during this period was an increase in B cell lymphoma and a relative decrease in T cell lymphoma and precursor lymphoid neoplasm. This change may be due to an increase in population age as well as other unknown factors. In support of this speculation, the median age of lymphoma patients in our cohort increased from 51 years in 1997–2006 to 56 years in 2007–2016. Increased plasma cell neoplasm was also notable, and an aging population and increasing body size may partly explain this increase. Obesity, a risk factor for plasma cell neoplasms, is steadily increasing in Korea.^[32,38]^ Other B cell lymphomas, including follicular lymphoma and mantle cell lymphoma, increased significantly. In addition to these changes associated with an increased aging population, our data show that infectious agent-associated lymphoma is decreasing. Extranodal NK/T cell lymphoma is a prototype of EBV-associated disease and virtually all tumor cells are infected by EBV. EBV-associated lymphoid malignancy is more prevalent in certain parts of Asia and Latin America, strongly suggesting genetic or environmental predisposition in the development of EBV-positive lymphoma. As noted, as socioeconomic conditions have improved in Korea, the age of first EBV infection is increasing like Western countries.^[33,39]^ EBV infection in young children whose immune system has not matured can lead to diseases such as chronic active EBV infection when they have genetic susceptibility. Although the role of EBV in the pathogenesis of extranodal NK/T cell lymphoma is not well known, the epidemiologic distribution of chronic active EBV infection and NK/T cell lymphoma is similar, suggesting that similar mechanisms may play a role in the development of NK/T cell lymphoma. Considering that, the decrease in the relative frequency of extranodal NK/T cell lymphoma in our cohort may be explained by the increasing of the age of first EBV infection, although we cannot exclude the influence of other risk factors. On the other hand, ENMZL has an etiologic relationship with H. pylori, autoimmune disease, and other infectious agents. Decrease in H. pylori infection in the general population may contribute to slowing the increase in ENMZL.

Malignant lymphoma is generally more common in male, but some subtypes have a female predominance. Typically, mediastinal large B cell lymphomas and subcutaneous panniculitis–like T cell lymphoma occur in male patients more frequently, which is also the case with our cohort.^[40]^ In this study, low grade follicular lymphoma was more prevalent in female but high grade lymphoma in males. In Western population, the vast majority of follicular lymphoma is of low grade and has a male to female ratio of 1:1.7.^[41,42]^ High grade follicular lymphoma by the 2008 WHO classification is heterogeneous lymphoid neoplasm which includes large B cell lymphoma with IRF-4 rearrangement, pediatric follicular lymphoma, and follicular lymphoma, grade 3B.^[43]^ It is different from low grade follicular lymphoma in the clinical, genetics, and even in the gender distribution. Extranodal marginal zone lymphoma has been known to show an equal distribution of gender or slight female predominance.^[44]^ In our cohort, extranodal marginal zone lymphoma affected more females than males and the gastric marginal zone lymphoma accounted for 61% of cases. Extragastric marginal zone lymphomas of the thyroid and salivary gland associated with autoimmune disease affect mainly female patients.^[45]^ In this study marginal zone lymphoma involving thyroid and salivary gland accounted for only a minority of cases.

The main drawback of this study is that the data are based on the patients from a single institution, so it is doubtful whether the results could reflect the nationwide data. In Korea, patients with lymphoma tend to gather in 4–5 large hospitals located in Seoul from each province, and Samsung Medical Center is one of these large hospitals. Therefore, the distribution of patients is not biased toward any particular type and may be consistent with the distribution of patients across the country. In addition, this study has several strengths compared with other previous studies. It included all lymphoid neoplasms in the liquid phase as well as solid phase, study subjects are the large-scale population and covers approximately 10% of all lymphoma patients in the country, lymphoma classification was performed based on sufficient ancillary studies by experienced hematopathologists, and the data is that of the most up-to-date. Despite the weaknesses of this study, these strengths will add value for future reference. In summary, we report herein the changing trends in the proportion of subtypes of malignant lymphoma in Korea and discuss the relationships with the population structure based on age and the prevalence of infectious agents. Considering the complexities of lifestyles and genetic factors that impact lymphomagenesis, the risk factors noted here may be somewhat superficial. Systematic epidemiological studies exploring these risk factors are needed to predict future changes in lymphoma frequency and to establish management strategies.

## Acknowledgments

We thank Dr. In-Suk Shon for statistical analysis.

## Author contributions

**Data curation:** Takuya Takayama, Junhun Cho, Seok Jin Kim, Won Seog Kim, Howe J. Ree.

**Funding acquisition:** Young Hyeh Ko.

**Methodology:** Jongmin Sim.

**Resources:** Junhun Cho.

**Supervision:** Young Hyeh Ko.

**Visualization:** Young Hyeh Ko.

**Writing – original draft:** Jongmin Sim.

**Writing – review & editing:** Young Hyeh Ko.

Jongmin Sim: 0000-0002-7106-1279.

Jongmin Sim orcid: 0000-0002-7106-1279.

## Supplementary Material

Supplemental Digital Content
