# Supplementary material for: Changing trends in lymphoid neoplasm distribution in South Korea: analysis of 8615 cases from a single institute, 1997–2016: An observational study
Source: Medicine (Baltimore). 2019 Nov 11;98(45):e17641. doi: 10.1097/MD.0000000000017641 (PMC6855639; doi:10.1097/MD.0000000000017641)
Supplement: Supplemental Digital Content [file medi-98-e17641-s001.doc]

Supplementary table 1. Biopsy sites and subtype distribution (continued next page).

|  | Bone | BM | Brain | LN | Eye | Stomach | SI | LI | Mediastinum |
| --- | --- | --- | --- | --- | --- | --- | --- | --- | --- |
| Other B-cell L | 2(1.0%) | 88(3.9%) | 4(1.3%) | 144(8.1%) | 1(0.4%) | 4(0.3%) | 3(1.4%) | 2(0.8%) | 4(2.5%) |
| Lymphoblastic leukemia | 11(5.4%) | 779(34.9%) | 4(1.3%) | 62(3.5%) | 1(0.4%) | 2(0.2%) | 2(0.9%) | 0 | 48(30.4%) |
| CLL/SLL | 0 | 122(5.5%) | 0 | 33(1.9%) | 0 | 0 | 0 | 1(0.4%) | 0 |
| Plasma cell N | 160(78%) | 1076(48.2%) | 4(1.3%) | 8(0.5%) | 3(1.1%) | 3(0.2) | 2(0.9%) | 1(0.4%) | 2(1.3%) |
| Extranodal MZL | 0 | 4(0.2%) | 3(1.0%) | 10(0.6%) | 251(89.6%) | 891(72.5%) | 23(10.6%) | 39(15%) | 7(4.4%) |
| Follicular L | 4(2%) | 4(0.2%) | 0 | 268(15.1%) | 7(2.5%) | 3(0.2%) | 25(11.5%) | 6(2.3%) | 1(0.6%) |
| Mantle cell L | 0 | 16(0.7%) | 0 | 57(3.2%) | 3(1.1%) | 14(1.1%) | 8(3.7%) | 16(6.2%) | 1(0.6%) |
| DLBCL | 20(9.8%) | 56(2.5%) | 282(91.6%) | 588(33.1%) | 9(3.2%) | 269(21.9%) | 109(50%) | 149(57.3%) | 50(31.6%) |
| Burkitt L | 2(1%) | 15(0.7%) | 2(0.6%) | 35(2%) | 1(0.4%) | 18(1.5%) | 19(8.7%) | 17(6.5%) | 0 |
| Aggressive  NK cell leukemia | 0 | 21(0.9%) | 0 | 0 | 0 | 0 | 0 | 0 | 0 |
| Extranodal  NK/T cell L | 0 | 9(0.4%) | 0 | 6(0.3%) | 2(0.7%) | 2(0.2%) | 5(2.3%) | 10(3.8%) | 0 |
| AITL | 0 | 1(0) | 0 | 144(8.1%) | 0 | 0 | 0 | 0 | 0 |
| ALCL | 4(2%) | 2(0.1%) | 3(1.0%) | 63(3.6%) | 2(0.7%) | 2(0.2%) | 1(0.5%) | 3(1.2% | 1(0.6%) |
| PTCL, NOS | 0 | 12(0.5%) | 6(1.9%) | 103(5.8%) | 0 | 19(1.5%) | 7(3.2%) | 5(1.9%) | 5(3.2%) |
| Hodgkin L | 2(1.0%) | 5(0.2%) | 0 | 243(13.7%) | 0 | 1(0.1%) | 0 | 0 | 39(24.7%) |
| Other T-cell L | 0 | 23(1%) | 0 | 10(0.6%) | 0 | 1(0.1%) | 14(6.4%) | 11(4.2%) | 0 |
| Total | 19 | 2233 | 308 | 1774 | 280 | 1229 | 218 | 260 | 158 |

ADT: aerodigestive tract, AITL: angioimmunoblastic T-cell lymphoma, ALCL: anaplastic large cell lymphoma, BM: bone marrow, Burkitt L: Burkitt lymphoma, CLL/SLL: Chronic lymphocytic leukemia/small lymphocyhtic lymphoma, DLBCL: diffuse large B-cell lymphoma, Entranodal NK/T cell L: Extranodal NK/T-cell lymphoma, Follicular L: Follicular lymphoma, Hodgkin L: Hodgkin lymphoma, LI: large intestine, Mantle cell L: Mantle cell lymphoma, MZL: marginal zone lymphoma, NK: nature killer, Other B-cell L: Other B-cell lymphoma, Other T-cell L: Other T-cell lymphoma, Plasma cell N: plasma cell neoplasms, PTCL, NOS: peripheral T-cell lymphoma, not otherwise specified, SI: small intestine, WR: Waldeyer's ring

(continued)

|  | Lung | Upper ADT | Nasal cavity | WR | Others | Skin | Breast | Kidney | Liver | Testis |
| --- | --- | --- | --- | --- | --- | --- | --- | --- | --- | --- |
| Other B-cell L | 12(6.5%) | 2(1.1%) | 6(2.1%) | 2(0.8%) | 26(4.6%) | 4(1.6%) | 0 | 5(15.6%) | 8(10.8%) | 0 |
| Lymphoblastic leukemia | 7(3.8%) | 3(1.6%) | 0 | 5(2.1%) | 13(2.3%) | 8(3.1%) | 1(1.8%) | 2(6.3%) | 1(1.4%) | 4(7.5%) |
| CLL/SLL | 0 | 0 | 0 | 1(0.4%) | 2(0.4%) | 0 | 0 | 1(3.1%) | 0 | 0 |
| Plasma cell N | 14(7.5%) | 14(7.4%) | 17(5.8%) | 3(1.3%) | 56(9.8%) | 5(2%) | 1(1.8%) | 1(3.1%) | 2(2.7%) | 7(13.2%) |
| Extranodal MZL | 75(40.3%) | 19(10.1%) | 4(1.4%) | 6(2.5%) | 86(15.1%) | 25(9.8%) | 2(3.5%) | 6(18.8%) | 0 | 0 |
| Follicular L | 1(0.5%) | 3(1.6%) | 1(0.3%) | 35(14.6%) | 35(6.1%) | 3(1.2%) | 3(5.3%) | 1(3.1%) | 0 | 0 |
| Mantle cell L | 0 | 5(2.7%) | 0 | 12(5%) | 4(0.7%) | 0 | 0 | 0 | 0 | 0 |
| DLBCL | 45(24.2%) | 90(47.9%) | 36(12.3%) | 138(57.7%) | 246(43.2%) | 21(8.2%) | 41(71.9%) | 13(40.6%) | 38(51.4%) | 36(67.9%) |
| Burkitt L | 0 | 6(3.2%) | 1(0.3%) | 7(2.9%) | 30(5.3%) | 0 | 2(3.5%) | 3(9.4%) | 5(6.8%) | 0 |
| Aggressive  NK cell leukemia | 0 | 0 | 0 | 0 | 0 | 0 | 0 | 0 | 0 | 0 |
| Extranodal  NK/T cell L | 1(0.5%) | 35(18.6%) | 221(75.7%) | 5(2.1%) | 24(4.2%) | 36(14.1%) | 0 | 0 | 5(6.8%) | 4(7.5%) |
| AITL | 0 | 0 | 0 | 2(0.8%) | 0 | 0 | 0 | 0 | 0 | 0 |
| ALCL | 6(3.2%) | 3(1.6%) | 2(0.8%) | 1(0.4%) | 20(3.5%) | 8(3.1%) | 3(5.3%) | 0 | 1(1.4%) | 1(1.9%) |
| PTCL, NOS | 6(3.2%) | 6(3.2%) | 3(1.0%) | 19(7.9%) | 9(1.6%) | 7(2.7%) | 2(3.5%) | 0 | 8(10.8%) | 0 |
| Hodgkin L | 19(10.2%) | 1(0.2%) | 1(0.3%) | 3(1.3%) | 9(1.6%) | 0 | 1(1.8%) | 0 | 2(2.7%) | 0 |
| Other T-cell L | 0 | 1(0.1%) | 0 | 0 | 10(1.8%) | 139(54.3%) | 1(1.8%) | 0 | 4(5.4%) | 0 |
| Total | 186 | 188 | 292 | 239 | 570 | 256 | 57 | 32 | 74 | 52 |

Supplementary table 2. Subtype distribution according to age group

| Subtypes\Age (year) | 0-9 | 10-19 | 20-29 | 30-39 | 40-49 | 50-59 | 60-69 | 70-79 | 80-89 | 90-99 |
| --- | --- | --- | --- | --- | --- | --- | --- | --- | --- | --- |
| Other B-cell L | 2(0.4%) | 18(4.6%) | 26(5.5%) | 29(3.8%) | 37(2.8%) | 53(2.7%) | 76(4%) | 61(5.5%) | 14(5.7%) | 1(6.7%) |
| Lymphoblastic  leukemia | 384(82.4%) | 211(53.7%) | 84(17.9%) | 94(12.2%) | 77(5.9%) | 53(2.7%) | 36(1.9%) | 13(1.2%) | 1(0.4%) | 0 |
| CLL/SLL | 0 | 0 | 0 | 7(0.9%) | 17(1.3%) | 41(2.1%) | 55(2.9%) | 31(2.8%) | 7(2.9%) | 2(13.3%) |
| Plasma cell N | 0 | 0 | 5(1.1%) | 43(5.6%) | 166(12.6%) | 382(19.7%) | 485(25.8%) | 250(22.4%) | 46(18.9%) | 2(13.3%) |
| Extranodal MZL | 0 | 10(2.5%) | 40(8.5%) | 148(19.1%) | 327(24.8%) | 433(22.3%) | 300(15.9%) | 172(15.4%) | 21(8.6%) | 0 |
| Follicular L | 1(0.2%) | 4(1%) | 7(1.5%) | 68(8.8%) | 89(6.8%) | 109(5.6%) | 75(4%) | 36(3.2%) | 10(4.1%) | 1(6.7%) |
| Mantle cell L | 0 | 0 | 0 | 2(0.3%) | 9(0.7%) | 31(1.6%) | 54(2.9%) | 36(3.2%) | 4(1.6%) | 0 |
| DLBCL | 13(2.8%) | 37(9.4%) | 96(20.4%) | 175(22.6%) | 358(27.2%) | 538(27.7%) | 546(29%) | 369(33.1%) | 99(40.6%) | 5(33.3%) |
| Burkitt L | 41(8.8%) | 23(5.9%) | 12(2.6%) | 14(1.8%) | 13(1%) | 18(0.9%) | 29(1.5%) | 8(0.7%) | 5(2.0%) | 0 |
| Aggressive  NK cell leukemia | 0 | 1(0.3%) | 5(1.1%) | 3(0.4%) | 6(0.5%) | 4(0.2%) | 1(0.1%) | 1(0.1%) | 0 | 0 |
| Extranodal  NK/T cell L | 1(0.2%) | 4(1%) | 25(5.3%) | 49(6.3%) | 87(6.6%) | 98(5%) | 66(3.5%) | 27(2.4%) | 7(2.9%) | 1(6.7%) |
| AITL | 0 | 0 | 2(0.4%) | 3(0.4%) | 12(0.9%) | 38(2.0%) | 38(2.0%) | 41(3.7%) | 13(5.3%) | 0 |
| ALCL | 14(3%) | 23(5.9%) | 21(4.5%) | 16(2.1%) | 12(0.9%) | 14(0.7%) | 17(0.9%) | 7(0.6%) | 2(0.8%) | 0 |
| PTCL, NOS | 2(0.4%) | 5(1.3%) | 12(2.6%) | 24(3.1%) | 37(2.8%) | 48(2.5%) | 46(2.4%) | 35(3.1%) | 8(3.3%) | 1(6.7%) |
| Hodgkin L | 5(1.1%) | 39(9.9%) | 94(20%) | 65(8.4%) | 40(3%) | 37(1.9%) | 28(1.5%) | 16(1.4%) | 4(1.6%) | 1(6.7%) |
| Other T-cell L | 3(0.6%) | 18(4.6%) | 41(8.7%) | 33(4.3%) | 29(2.2%) | 45(2.3%) | 29(1.5%) | 12(1.1%) | 3(1.2%) | 1(6.7%) |
| Total | 466(100%) | 393(100%) | 470(100%) | 773(100%) | 1316(100%) | 1942(100%) | 1881(100%) | 1115(100%) | 244(100%) | 15(100%) |

AITL: angioimmunoblastic T-cell lymphoma, ALCL: anaplastic large cell lymphoma, Burkitt L: Burkitt lymphoma, CLL/SLL: Chronic lymphocytic leukemia/small lymphocyhtic lymphoma, DLBCL: diffuse large B-cell lymphoma, Entranodal NK/T cell L: Extranodal NK/T-cell lymphoma, Follicular L: Follicular lymphoma, Hodgkin L: Hodgkin lymphoma, Mantle cell L: Mantle cell lymphoma, MZL: marginal zone lymphoma, NK: nature killer, Other B-cell L: Other B-cell lymphoma, Other T-cell L: Other T-cell lymphoma, Plasma cell N: plasma cell neoplasms, PTCL, NOS: peripheral T-cell lymphoma, not otherwise specified

Supplementary table 3. Subtype distribution according to age group in male patients.

| Subtypes\Age (year) | 0-9 | 10-19 | 20-29 | 30-39 | 40-49 | 50-59 | 60-69 | 70-79 | 80-89 | 90-99 |
| --- | --- | --- | --- | --- | --- | --- | --- | --- | --- | --- |
| Other B-cell L | 2(0.7%) | 17(6.8%) | 16(6.3%) | 19(4.5%) | 26(3.6%) | 30(2.8%) | 45(4.2%) | 37(5.7%) | 10(7%) | 0 |
| Lymphoblastic  leukemia | 212(78.2%) | 134(53.4%) | 54(21.2%) | 50(11.9%) | 33(4.4%) | 24(2.2%) | 27(1.6%) | 8(1.2%) | 1(0.7%) | 0 |
| CLL/SLL | 0 | 0 | 0 | 4(1%) | 12(1.6%) | 24(2.2%) | 34(3.2%) | 20(3.1%) | 4(2.8%) | 1(10%) |
| Plasma cell N | 0 | 0 | 3(1.2%) | 29(6.9%) | 104(14%) | 223(20.9%) | 261(24.5%) | 135(20.7%) | 29(20.4%) | 2(20%) |
| Extranodal MZL | 0 | 1(0.4%) | 12(4.7%) | 69(16.5%) | 163(21.9%) | 191(17.9%) | 136(12.8%) | 98(15.1%) | 6(4.2%) | 0 |
| Follicular L | 0 | 4(1.6%) | 2(0.8%) | 27(6.4%) | 49(6.6%) | 62(5.8%) | 37(3.5%) | 20(3.1%) | 8(5.6%) | 1(10%) |
| Mantle cell L | 0 | 0 | 0 | 2(0.5%) | 8(1.1%) | 24(2.2%) | 40(3.8%) | 26(4.0%) | 4(2.8%) | 0 |
| DLBCL | 5(1.8%) | 23(9.2%) | 50(19.6%) | 90(21.5%) | 200(26.9%) | 301(28.2%) | 319(30%) | 208(32%) | 56(39.4%) | 2(20%) |
| Burkitt L | 38(12.5%) | 18(7.2%) | 10(3.9%) | 12(2.9%) | 7(0.9%) | 12(1.1%) | 21(2%) | 4(0.6%) | 3(2.1%) | 0 |
| Aggressive  NK cell leukemia | 0 | 1(0.4%) | 4(1.6%) | 3(0.7%) | 5(0.7%) | 1(0.1%) | 0 | 0 |  | 0 |
| Extranodal  NK/T cell L | 1(0.4%) | 2(0.8%) | 15(5.9%) | 37(8.8%) | 54(7.3%) | 59(5.5%) | 46(4.3%) | 19(2.9%) | 4(2.8%) | 1(10%) |
| AITL | 0 | 0 | 0 | 1(0.2%) | 9(1.2%) | 24(2.2%) | 28(2.6%) | 28(4.3%) | 6(4.2%) | 0 |
| ALCL | 10(3.7%) | 11(4.4%) | 17(6.7%) | 11(2.6%) | 7(0.9%) | 10(0.9%) | 12(1.1%) | 4(0.6%) | 2(1.4%) | 0 |
| PTCL, NOS | 2(0.7%) | 5(2%) | 6(2.4%) | 14(3.3%) | 26(3.5%) | 33(3.1%) | 28(2.6%) | 24(3.7%) | 5(3.5%) | 1(10%) |
| Hodgkin L | 5(1.8%) | 28(11.2%) | 41(16.1%) | 35(8.4%) | 27(3.6%) | 32(3%) | 24(2.3%) | 13(2%) | 3(2.1%) | 1(10%) |
| Other T-cell L | 0 | 7(2.8%) | 25(9.8%) | 16(3.8%) | 13(1.7%) | 18(1.7%) | 16(1.5%) | 7(1.1%) | 1(0.7%) | 1(10%) |
| Total | 271(100%) | 251(100%) | 255(100%) | 419(100%) | 743(100%) | 1068(100%) | 1064(100%) | 651(100%) | 142(100%) | 10(100%) |

AITL: angioimmunoblastic T-cell lymphoma, ALCL: anaplastic large cell lymphoma, Burkitt L: Burkitt lymphoma, CLL/SLL: Chronic lymphocytic leukemia/small lymphocyhtic lymphoma, DLBCL: diffuse large B-cell lymphoma, Entranodal NK/T cell L: Extranodal NK/T-cell lymphoma, Follicular L: Follicular lymphoma, Hodgkin L: Hodgkin lymphoma, Mantle cell L: Mantle cell lymphoma, MZL: marginal zone lymphoma, NK: nature killer, Other B-cell L: Other B-cell lymphoma, Other T-cell L: Other T-cell lymphoma, Plasma cell N: plasma cell neoplasms, PTCL, NOS: peripheral T-cell lymphoma, not otherwise specified

Supplementary table 4. Subtype distribution according to age group in female patients.

| Subtypes\Age (year) | 0-9 | 10-19 | 20-29 | 30-39 | 40-49 | 50-59 | 60-69 | 70-79 | 80-89 | 90-99 |
| --- | --- | --- | --- | --- | --- | --- | --- | --- | --- | --- |
| Other B-cell L | 0 | 1(0.7%) | 10(4.7%) | 10(2.8%) | 11(1.9%) | 23(2.6%) | 31(3.8%) | 24(5.2%) | 4(3.9%) | 1(20%) |
| Lymphoblastic  leukemia | 172(88.2%) | 77(54.2%) | 30(14%) | 44(12.4%) | 44(7.7%) | 29(3.3%) | 19(2.3%) | 5(1.1%) | 0 | 0 |
| CLL/SLL | 0 | 0 | 0 | 3(0.8%) | 5(0.9%) | 17(1.9%) | 21(2.6%) | 11(2.4%) | 3(2.9%) | 1(20%) |
| Plasma cell N | 0 | 0 | 2(0.9%) | 14(4%) | 62(10.8%) | 159(18.2%) | 224(27.4%) | 115(24.8%) | 17(16.7%) | 0 |
| Extranodal MZL | 0 | 9(6.3%) | 28(13.1%) | 79(22.3%) | 164(28.6%) | 242(27.7%) | 164(20.1%) | 74(15.9%) | 15(14.7%) | 0 |
| Follicular L | 1(0.5%) | 0 | 5(2.3%) | 41(11.6%) | 40(7.0%) | 47(5.4%) | 38(4.7%) | 16(3.4%) | 2(2.0%) | 0 |
| Mantle cell L | 0 | 0 | 0 | 0 | 1(0.2%) | 7(0.8%) | 14(1.7%) | 10(2.2%) | 0 | 0 |
| DLBCL | 8(4.1%) | 14(9.9%) | 46(21.5%) | 85(24%) | 158(27.6%) | 237(27.1%) | 227(27.8%) | 161(34.7%) | 43(42.2%) | 3(60%) |
| Burkitt L | 7(3.6%) | 5(3.5%) | 2(0.9%) | 2(0.6%) | 6(1%) | 6(0.7%) | 8(1%) | 4(0.9%) | 2(2%) | 0 |
| Aggressive  NK cell leukemia | 0 | 0 | 1(0.5%) | 0 | 1(0.2%) | 3(0.3%) | 1(0.1%) | 1(0.2%) | 0 | 0 |
| Extranodal  NK/T cell L | 0 | 2(1.4%) | 10(4.7%) | 12(3.4%) | 33(5.8%) | 39(4.5%) | 20(2.4%) | 8(1.7%) | 3(2.9%) | 0 |
| AITL | 0 | 0 | 2(0.9%) | 2(0.6%) | 3(0.5%) | 14(1.6%) | 10(1.2%) | 13(2.8%) | 7(6.9%) | 0 |
| ALCL | 4(2.1%) | 12(8.5%) | 4(1.9%) | 5(1.4%) | 5(0.9%) | 4(0.5%) | 5(0.6%) | 3(0.7%) | 0 | 0 |
| PTCL, NOS | 0 | 0 | 6(2.8%) | 10(2.8%) | 11(1.9%) | 15(1.7%) | 18(2.2%) | 11(2.4%) | 3(2.9%) | 0 |
| Hodgkin L | 0 | 11(7.7%) | 52(24.2%) | 30(8.5%) | 13(2.3%) | 5(0.6%) | 4(0.5%) | 3(0.7%) | 1(1%) | 0 |
| Other T-cell L | 3(1.5%) | 11(7.7%) | 16(7.5%) | 17(4.8%) | 16(2.8%) | 27(3.1%) | 13(1.6%) | 5(1.1%) | 2(2%) | 0 |
| Total | 195(100%) | 142(100%) | 214(100%) | 354(100%) | 573(100%) | 874(100%) | 817(100%) | 464(100%) | 102(100%) | 5(100%) |

AITL: angioimmunoblastic T-cell lymphoma, ALCL: anaplastic large cell lymphoma, Burkitt L: Burkitt lymphoma, CLL/SLL: Chronic lymphocytic leukemia/small lymphocyhtic lymphoma, DLBCL: diffuse large B-cell lymphoma, Entranodal NK/T cell L: Extranodal NK/T-cell lymphoma, Follicular L: Follicular lymphoma, Hodgkin L: Hodgkin lymphoma, Mantle cell L: Mantle cell lymphoma, MZL: marginal zone lymphoma, NK: nature killer, Other B-cell L: Other B-cell lymphoma, Other T-cell L: Other T-cell lymphoma, Plasma cell N: plasma cell neoplasms, PTCL, NOS: peripheral T-cell lymphoma, not otherwise specified
